# Supplementary material for: Expression of endogenous Anopheles gambiae microRNAs using an Anopheles gambiae densovirus (AgDNV) intronic expression system
Source: Parasit Vectors. 2025 Aug 19;18:355. doi: 10.1186/s13071-025-06994-7 (PMC12366140; doi:10.1186/s13071-025-06994-7)
Supplement: Supplementary file 1 — Supplementary Material 1. [file 13071_2025_6994_MOESM1_ESM.docx]

**Expression of endogenous *Anopheles gambiae* microRNAs using an *Anopheles gambiae* densovirus (AgDNV) intronic expression system**

**Supplemental information**

| **Construct** | **Intronic sequences** | **Mature miRNA sequences** |
| --- | --- | --- |
| ***miR8*** | **gtaagtgcgc**ttgccactcattcctgcaggGGGTGTCTGTTCACATCTTACCGGGCAGCATTAGATATGTTATCGGATATTTCTAATACTGTCAGGTAAAGATGTCGTCCGAGCCCagatctagtggatggagtcatcaactattggtct**tactgacatccactttgcctttctctccacag** | uaauacugucagguaaagauguc |
| ***miR34*** | **gtaagtgcgc**ttgccactcattcctgcaggGAGGCAATATACGCTCTGGCAGTGTGGTTAGCTGGTTGTGTGGTTTTCCCATCTTCACAGCCACTATCCGCCCTGCCGTCGCGCTAATGCagatctagtggatggagtcatcaactattggtct**tactgacatccactttgcctttctctccacag** | uggcagugugguuagcuggu |
| ***miR305*** | **gtaagtgcgc**ttgccactcattcctgcaggTTTGTCACATGTCTATTGTACTTCATCAGGTGCTCTGGTGGATTTGAGAAAACCCGGCACATGTTGGAGTACACTCTATGTGCTGACAAGagatctagtggatggagtcatcaactattggtc*t****tactgacatccactttgcctttctctccacag*** | auuguacuucaucaggugcucug |
| ***miR375*** | **gtaagtgcgc**ttgccactcattcctgcaggTGTTGCGATGAGACAGAATTTGGATTACTTAAGCCACGCGTACAGAAACTTTTTAGAATGAAAGAGTTTGTTCGTTTGGCTCGAGTTATGCCGGTTCTAAATTGCAGCAagatctagtggatggagtcatcaactattggtct**tactgacatccactttgcctttctctccacag** | uuuguucguuuggcucgaguua |
| ***miR8SP*** | **gtaagtgcgc**GACATCTTTACACTCAGTATTAcgcgGACATCTTTACACTCAGTATTAcgcgGACATCTTTACACTCAGTATTAcgcgGACATCTTTACACTCAGTATTAcgcgGACATCTTTACACTCAGTATTAcgcgGACATCTTTACACTCAGTATTAcgcgGACATCTTTACACTCAGTATTAcgcgGACATCTTTACACTCAGTATTAcgcgGACATCTTTACACTCAGTATTAcgcgGACATCTTTACACTCAGTATTA**tactgacatccactttgcctttctctccacag** | NA |
| ***NS RNA*** | **gtaagtgcgc**ttgccactcattcctgcaggATAGAAACTACACCATTAACCTTCCTGAGAACCGGGAGGTGGGAATCCGTCACATATGAGAAGGTATTTGCCCGATAATCAATACTCCAGGCTTCTAACTagatctagtggatggagtcatcaactattggtct**tactgacatccactttgcctttctctccacag** | NA |

**Table S1: Intronic sequences for transducing constructs.** Bold font indicates the intronic segments of the splice donor and splice acceptor site. Uppercase font indicates the pre-miRNA sequences for miRNAs (*miR8*, *miR34*, *miR305*, or *miR375*), or the miRNA sponge blocks that are the reverse complement of *miR8* (*miR8SP*), or the *nonsense RNA* sequence (*NS RNA*). Lowercase, non-bold font indicates spacer sequences. Mature miRNA sequences are given for *miR8*, *miR34*, *miR305*, and *miR375*.

| **Replicate** | **Treatment** | **Transducing concentration (copies/ul)** | **Wild-type concentration (copies/ul)** | **Transducing amount injected (copies)** | **Wild-type amount injected (copies)** |
| --- | --- | --- | --- | --- | --- |
| **Replicate 1** | ***SA*** | 2.62 x 10^5^ | 1.04 x 10^7^ | 2.0 x 10^6^ | 7.9 x 10^7^ |
|  | ***SD*** | 3.31 x 10^5^ | 1.41 x 10^7^ | 2.0 x 10^6^ | 8.5 x 10^7^ |
|  | ***miR8*** | 2.07 x 10^5^ | 3.14 x 10^7^ | 2.0 x 10^6^ | 3.0 x 10^8^ |
|  | ***miR8SP*** | 6.35 x 10^5^ | 8.92 x 10^6^ | 2.0 x 10^6^ | 2.7 x 10^7^ |
|  | ***miR34*** | 5.25 x 10^5^ | 8.00 x 10^6^ | 2.0 x 10^6^ | 3.0 x 10^7^ |
|  | ***miR305*** | 5.58 x 10^5^ | 8.32 x 10^6^ | 2.0 x 10^6^ | 3.0 x 10^7^ |
|  | ***miR375*** | 3.39 x 10^5^ | 1.92 x 10^7^ | 2.0 x 10^6^ | 1.1 x 10^8^ |
|  | ***NS*** | 4.59 x 10^5^ | 8.44 x 10^6^ | 2.0 x 10^6^ | 3.6 x 10^7^ |
| **Replicate 2** | ***miR34*** | 7.14 x 10^7^ | 9.39 x 10^8^ | 1.4 x 10^7^ | 1.9 x 10^8^ |
|  | ***miR375*** | 6.15 x 10^7^ | 3.77 x 10^8^ | 1.2 x 10^7^ | 7.5 x 10^7^ |
|  | ***NS*** | 9.26 x 10^7^ | 1.03 x 10^9^ | 1.9 x 10^7^ | 2.1 x 10^8^ |
| **Replicate 3** | ***miR34*** | 1.10 x 10^7^ | 6.14 x 10^7^ | 2.2 x 10^6^ | 1.2 x 10^7^ |
|  | ***miR375*** | 2.02 x 10^7^ | 6.88 x 10^7^ | 4.0 x 10^6^ | 1.4 x 10^7^ |
|  | ***NS*** | 1.63 x 10^7^ | 4.70 x 10^7^ | 3.3 x 10^6^ | 9.4 x 10^6^ |

**Table S2: Concentrations of transducing and WT virus used to inject mosquitoes.** Listed wild-type viral concentrations in copies/ul were found using qPCR against WT AgDNV *nonstructural protein 1* for vWTAgDNV and a standard curve of pWTAgDNV. For transducing virus concentrations, qPCR reactions against *EGFP* and standard curve of pAcEGFP was used to calculate copies/ul. Replicate 1 samples were concentrated to ~1 x 10^7^ copies/ul of the transducing virus using an Amicon Ultra 0.5 mL Ultracel 10K filters (UFC501096) before injection. Injection values for Replicate 1 reflect the concentrated amount injected whereas the transducing and wild-type concentration represent the copies/ul measured by qPCR before concentrating. Injected copies were calculated using an injection volume of 200 nl per mosquito. Replicate 2 and Replicate 3 viruses did not need to be concentrated before injection.

| **Name** | **Sequence** | **Source** |
| --- | --- | --- |
| **AnSwim-F** | TCAAGGATCGTGGAACGGTG |  |
| **AnSwim-R** | ATTGGCCCCAGGAGTTGATG |  |
| **MISO-F** | AGACGATGGAGGGACTGATG | Baldini et. al., 2013 |
| **MISO-R** | GGATTCGCTTTCGTGCTG | Baldini et. al., 2013 |
| **Cactus-F** | GAACGGCTGCGCTTTAACA | Frolet et. al., 2006 |
| **Cactus-R** | TCGTTCAAGTTCTGTGCAAGTGT | Frolet et. al., 2006 |
| **Rel1A-F** | TCAACAGATGCCAAAAGAGGAAAT | Frolet et. al., 2006 |
| **Rel1A-R** | CTGGTTGGAGGGATTGTG | Frolet et. al., 2006 |
| **Caspar-F** | CCGATCATCAATCAGCAGAA | League, et. al., 2017 |
| **Caspar-R** | GTGCAGATAGATCGCCAACA | League, et. al., 2017 |
| **Rel2-F** | ACCGATACGGAAAGTGTGCT | Meister et. al., 2005 |
| **Rel2-R** | CGGTGCTCCTCGTAATGACT | Meister et. al., 2005 |
| **APL1-F** | GCCTGATCCAACCATACATACCA | Frolet et. al., 2006 |
| **APL1-R** | GGCTGAGTGCTATGAGGTAAATGTAC | Frolet et. al., 2006 |
| **S7-F** | TCCTGGAGCTGGAGATGAAC | Dong et. al., 2006 |
| **S7-R** | GACGGGTCTGTACCTTCTGG | Dong et. al., 2006 |

**Table S3: Primers used for target gene qPCR reactions.** Primers were taken from the reported sources listed in the table except for primers targeting *Swim1* that were developed during this study.

| **Name** | **Sequence** |
| --- | --- |
| **miR8F** | TAATACTGTCAGGTAAAGATGTC |
| **miR34F** | TGGCAGTGTGGTTAGCTGGT |
| **miR305F** | ATTGTACTTCATCAGGTGCTCTG |
| **miR375F** | TTTGTTCGTTTGGCTCGAGTTA |
| **U6F** | ACTAAAATTGGAACGATACAG |

**Table S4: Primers used for miRNA quantification qPCR reactions.** Primers matching the sequence of each miRNA were developed against each mature miRNA sequence. The *U6* primer was developed against *An. gambiae* *U6* and this small RNA served as a reference with which to compare miRNA expression.

**
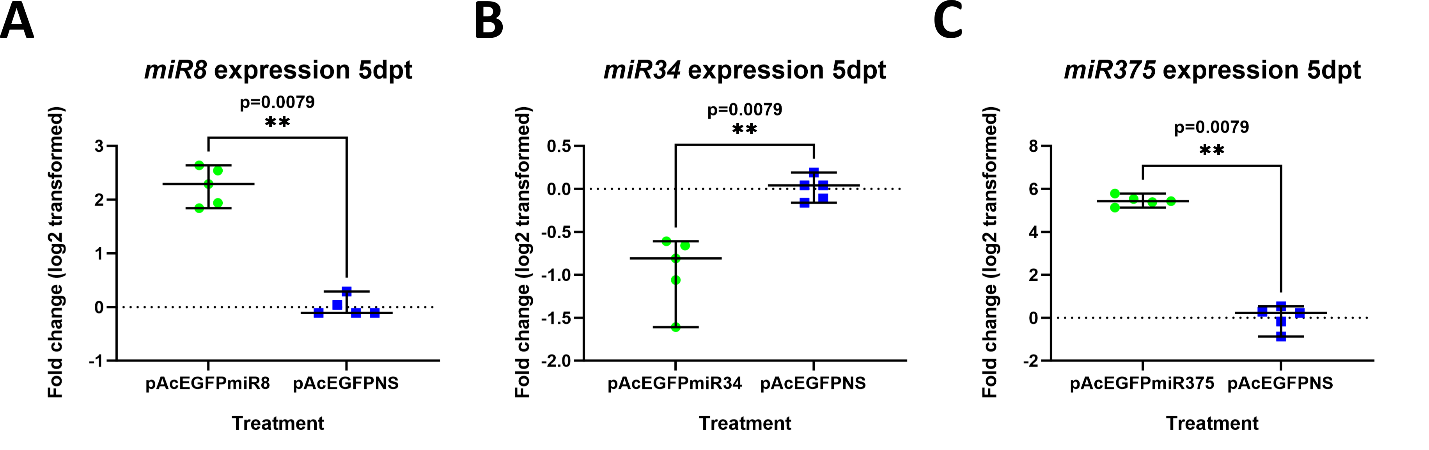
**

**Figure S1: Expression of *miR8*, *miR34*, and *miR375* in Sua5B cells 5 days post-transfection. A)** Levels of *miR8* were elevated when cells were co-transfected with pWTAgDNV and pAcEGFPmiR8. **B)** Levels of *miR34* were lower when cells were transfected with pWTAgDNV and pAcEGFPmiR34. **C)** miR375 expression was increased when cells were transfected with pWTAgDNV and pAcEGFPmiR375. Dashed line indicates a fold change of 0. Green dots represent individual wells of cells co-transfected with indicated miRNA-expressing plasmids and pWTAgDNV whereas blue squares indicate individual wells co-transfected with control pAcEGFPNS and pWTAgDNV. Data in all graphs did not follow a normal distribution as per D’Agostino-Pearson normality tests. Differences between groups were analyzed using two-tailed Mann-Whitney tests.


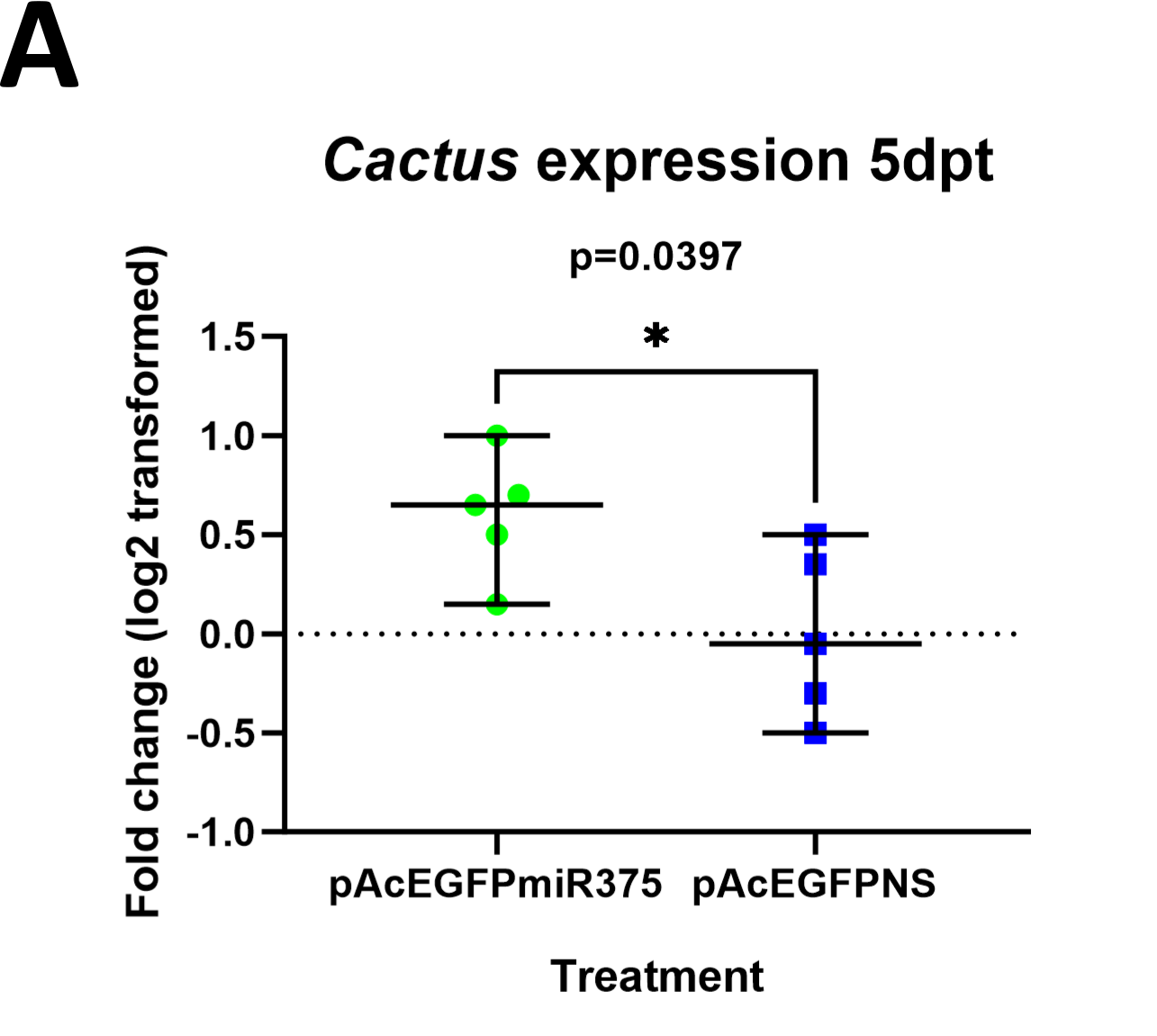


**Figure S2: Expression of *Cactus* in Sua5B cells 5 days post-transfection with pAcEGFPmiR375 and pAcEGFPNS. A)** *Cactus* transcript levels were elevated when cells were co-transfected with pWTAgDNV and pAcEGFPmiR375. Dashed line indicates a fold change of 0. Green dots represent individual wells of cells co-transfected with pAcEGFPmiR375 and pWTAgDNV whereas blue squares indicate individual wells co-transfected with control pAcEGFPNS and pWTAgDNV. Data failed a D’Agostino-Pearson normality test and was analyzed using a two-tailed Mann-Whitney test.


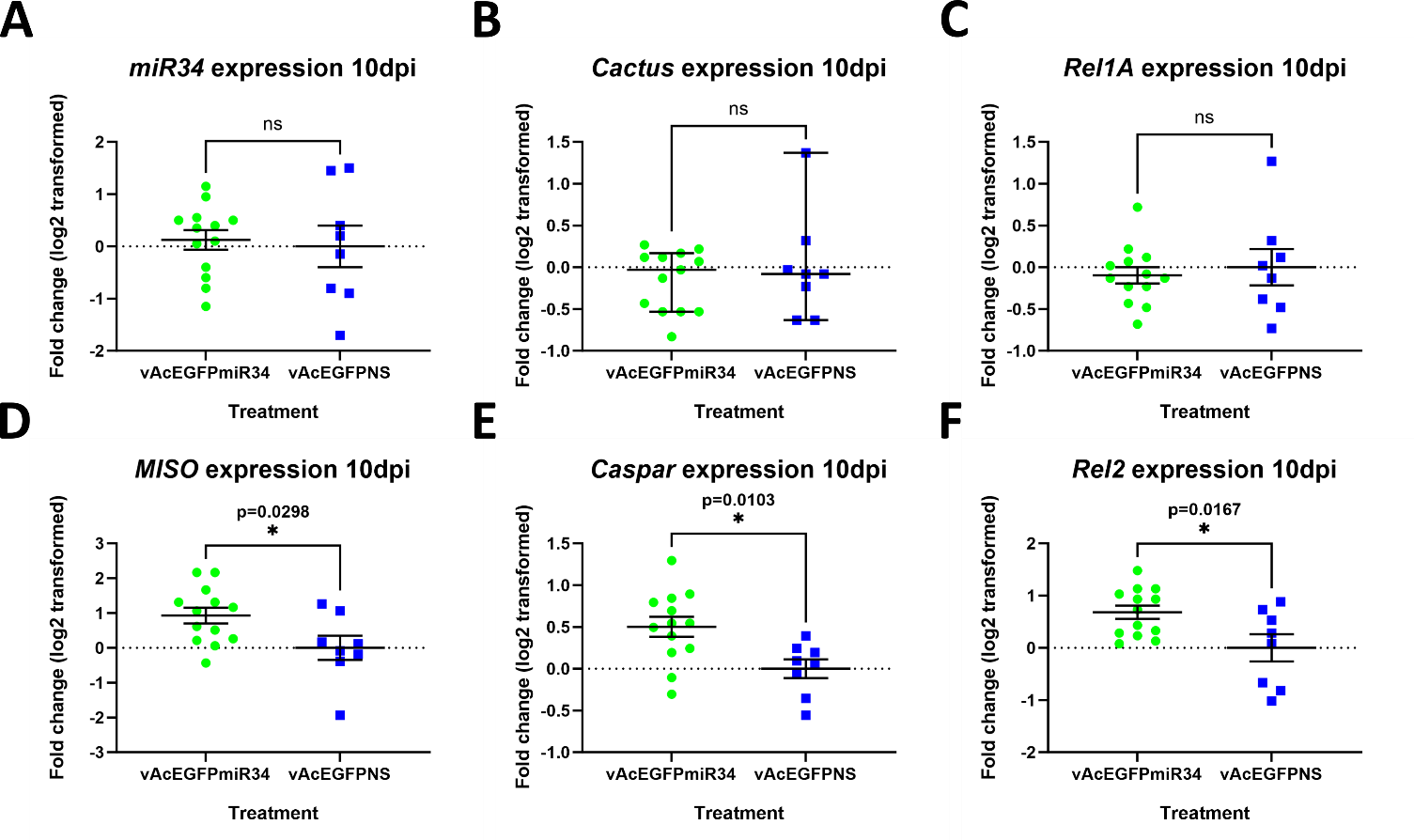


**Figure S3: Expression of *miR34*, and target gene transcripts in mosquitoes 10 days post-injection with vWTAgDNV and vAcEGFPmiR34. A)** Expression of *miR34* was unchanged in mosquitoes that were injected with vWTAgDNV and vAcEGFPmiR34. **B)** *Cactus* transcript levels were not altered following injection. **C)** Levels of *Rel1A* were also not changed 10 days post-injection. **D)** *MISO* levels were significantly elevated following injection. **E)** *Caspar* expression was enhanced in mosquitoes following injection of vWTAgDNV and vAcEGFPmiR34. **F)** *Rel2* expression was also enhanced in mosquitoes following injection. Dashed line indicates a fold change of 0. Green dots represent individual mosquitoes co-injected with vAcEGFPmiR34 and pWTAgDNV whereas blue squares indicate individual mosquitoes co-injected with control vAcEGFPNS and vWTAgDNV. Data in graphs A and C-F were normal as assessed by a D’Agostino-Pearson normality test and were analyzed using an unpaired two-tailed t-test. Data in graph B were not normal and were analyzed using a two-tailed Mann-Whitney test.
